# Bridging reproductive and microbial ecology: a case study in arbuscular mycorrhizal fungi

**DOI:** 10.1038/s41396-018-0314-7

**Published:** 2018-11-30

**Authors:** Carlos A. Aguilar-Trigueros, Stefan Hempel, Jeff R. Powell, William K. Cornwell, Matthias C. Rillig

**Affiliations:** 1 Freie Universität Berlin, Institute of Biology, Berlin, D-14195 Germany; 2 Berlin-Brandenburg Institute of Advanced Biodiversity Research (BBIB), Berlin, D-14195 Germany; 3 Hawkesbury Institute for the Environment, University of Western Sydney, Penrith, NSW 2751 Australia; 4 Evolution and Ecology Research Centre, School of Biological, Earth and Environmental Sciences, University of New South Wales, Sydney, NSW Australia

**Keywords:** Microbial ecology, Fungal ecology

## Abstract

Offspring size is a key trait for understanding the reproductive ecology of species, yet studies addressing the ecological meaning of offspring size have so far been limited to macro-organisms. We consider this a missed opportunity in microbial ecology and provide what we believe is the first formal study of offspring-size variation in microbes using reproductive models developed for macro-organisms. We mapped the entire distribution of fungal spore size in the arbuscular mycorrhizal (AM) fungi (subphylum Glomeromycotina) and tested allometric expectations of this trait to offspring (spore) output and body size. Our results reveal a potential paradox in the reproductive ecology of AM fungi: while large spore-size variation is maintained through evolutionary time (independent of body size), increases in spore size trade off with spore output. That is, parental mycelia of large-spored species produce fewer spores and thus may have a fitness disadvantage compared to small-spored species. The persistence of the large-spore strategy, despite this apparent fitness disadvantage, suggests the existence of advantages to large-spored species that could manifest later in fungal life history. Thus, we consider that solving this paradox opens the door to fruitful future research establishing the relationship between offspring size and other AM life history traits.

## Introduction

Offspring size is a highly variable trait in living organisms. For example, in birds, egg size ranges from 1.2 cm in length for hummingbirds to 14.4 cm for the common ostrich (major diameter length) [1]. In plants, seed size in terms of mass ranges from a tenth of a microgram in orchids to 20 kg in the double coconut [2]. In marine fish, egg size varies from 0.5 to 90 mm in diameter, a several million-fold difference in terms of volume [3]. This large variation is only partly explained by the size of the adults [4].

This large variation in offspring size is not random or inconsequential: it has important ecological and evolutionary meaning because offspring size—a proxy for the quantity of resources that parents allocate to each individual offspring—greatly influences the survival and fitness of the offspring [5, 6]. For example, in many taxa, bigger offspring usually have higher survival, including in fish [7], birds [8], insects [9], and plants [10]. Moreover, offspring size influences the fitness of the parents: the energy and resources allocated to offspring can reduce the fitness of the parents either directly (e.g., bigger offspring size usually translates into fewer offspring) or indirectly (reduced parent survival when the cost of reproduction is too high) [11]. Thus, from the perspective of inclusive fitness theory [12], offspring size is a particularly interesting trait because its variation is regulated by selection pressure acting on both the parental and progeny generations [9].

Despite the varied and extensive study of offspring size in different taxa and its implications, there are surprising omissions. The entire microbial world, for instance, seems to be overlooked: studies addressing offspring-size variation of microscopic fungi, bacteria, archaea, and protists are extremely rare [13]. One explanation for this oversight of reproductive ecology in the microbial world could be that offspring-size variation is irrelevant for such groups. While this explanation seems plausible at first sight, we think it is unlikely and instead believe that a better explanation for this omission is a combination of technological limitations for studying microbial reproduction and the limited communication between scientists studying microbial and reproductive ecology. For example, the ecological implications of spore size have received more attention among mycologists working with macro-fungi (groups with aboveground fruiting bodies), compared to microfungi [14, 15], probably because the macro-fungi are more easily directly observed.

Bridging microbial and reproductive ecology would bring many benefits. The first benefit would be a better appreciation within microbial ecology of how offspring size can be used as a functional trait reflecting the ecology and biogeographic patterns of different species [16]. Second, taking into account the particularities of the microbial world would provide researchers a chance to rethink, revise, and modify macroorganism-centric theories. It is possible that theories developed as a result of exclusively and carefully studying plants and animals are not applicable to the entire tree of life, including its microbial branches.

Here we provide what we believe to be the first formal study of offspring-size variation in the arbuscular mycorrhizal fungi (AM fungi, microbes involved in a nutrient-exchange symbiosis with the roots of almost 80% of plant species [17]). The lack of discussion about the causes and consequences of offspring-size variation in AM fungi contrasts with the well-documented observation that these fungi allocate considerable resources to reproduction during their lifecycle despite no documented evidence of sexual reproduction. Indeed, the asexual spores (known as “azygospores”) of some AM species are noted to be among the largest known in the fungal kingdom, while other AM species spend considerable biomass to enclose these asexual spores in sporocarps [18]. In this paper, we (1) summarize the currently scattered information on AM fungal spore variation; (2) map the phylogenetic distribution of AM fungal spore size; and (3) interpret the ecological meaning of this variation testing two cornerstone concepts in reproductive ecology: the trade-off with spore output and correlations with other life history traits. By addressing these three points, we show how reproductive ecological theory can be used to understand the ecology and evolution of microorganisms.

## Materials and methods

### Reproductive ecology models

We concentrate on testing two cornerstone concepts in reproductive ecology: first, whether there is a trade-off between offspring output and offspring size. This trade-off is expected to take the form of a Smith and Fretwell model (SFM) [4, 19] as represented in the following equation (Eq. 1):

1 $$R = \frac{A}{{W_0^b}}$$

For a given AM fungal species, *R* is the spore output (the number of spores produced in a given amount of time); *A* is the total amount of resources that a fungal species allocates to spore production; *W*_0_ is the size of an individual spore of the species; and *b* is the scaling factor in the relationship.

The second cornerstone concept we test is the allometric scaling of reproductive traits (*W*_0_, spore size and *R*, spore output) to total body size (fungal biomass produced). As seen in mammals and plants, this scaling also follows a power function of the form (Eq. 2):

2 $$RW_0 = \sigma W_\alpha ^\beta$$

where *W*_α_ is fungal body size, *σ* is a constant of proportionality, and *β* is a scaling factor.

### Data collection

#### Spore size

We created a fungal spore-trait database that includes spore sizes for all currently described 294 AM fungal species, following the taxonomy, as reported in Arthur Schüßler’s website (http://www.amf-phylogeny.com/, version from January 16th, 2017), which reflects the current consensus in Glomeromycotina phylogeny [20]. Size data were obtained from the original description of each species, or, when this source could not be accessed, from the International Culture Collection of (Vesicular) Arbuscular Mycorrhizal Fungi (https://invam.wvu.edu/) and the culture collection of Dr. Janusz Błaszkowski (http://www.zor.zut.edu.pl/Glomeromycota/Species%20descriptions%20of%20AMF.html).

These data sources report spore size as diameter ranges for each species. These ranges usually include an inner range within which most spores for a given species fall, and an outer range with extreme values (the actual number of spores used to determine this range is usually not given, but is roughly 100 spores in the few cases where it is). For completeness, we report both inner and outer ranges in the database (i.e., we include all documented data on spore size, see supplementary material) but, for our analyses, we calculated the mean of the two inner ranges and the mean of the two outer ranges. These values were used to independently provide a single-spore diameter per range, per species (i.e., we obtained spore diameters based on inner and outer ranges). Then, we used the diameter values from the inner and outer ranges, separately, to calculate spore size either as sphere volumes when spores are described predominantly as “globose” with only one diameter range provided, or as prolate spheroid volumes when described as predominantly “sub-globose” with two diameter ranges provided (the shortest diameter was duplicated to calculate volume, see supplementary material for calculations). In this way, each AM fungal species correspond to a unique spore size (volume) entry (except for six dimorphic species in the Ambisporaceae that have two different entries corresponding to each of their distinct spore types, i.e., the “acaulosporoid” and “glomoid” type).

For testing the trade-off of spore size to spore output and the allometric relationship between spore size and fungal body size (see below), we further converted biovolumes of spores into biomass using the conversion factor that 1 µm^3^ of a fungal spore weighs 3.64 × 10^−7^ µg. This conversion factor is based on the weight of 1000 spores of the AM fungus *Funneliformis caledonium* (syn. *Glomus caledonium*) as reported by Beilby and Kidby [21]. We use data from these authors to obtain this conversion factor because, to our knowledge, this is the only published reference where both spore volume and spore weight have been reported for a species of AMF (see supplementary material for more details on these conversions).

#### Phylogenetic tree

The phylogenetic tree for AM fungi is based on the ribosomal genes of the small subunit (SSU), the internal transcribed spacer region (ITS) and the large subunit (LSU), as suggested in refs. [22, 23]. First, the phylogenetic reference DNA sequences for the species in the trait table were extracted from the [22] dataset. This gave us DNA sequence data for 81 species from our spore size database. For the remaining species, we conducted a search in the NCBI Genbank database, both in the title and the organism identifiers due to occasional mismatches, and we restricted the search to sequences stemming directly from identified spores. We selected the longest DNA sequence entries with as much overlap to the region given in the [23] dataset. Thereby, we added sequence information for another 67 species for a total of 148 species, which represent 50% of all species in the trait dataset.

The sequences were aligned using MAFFT version 7 [24] by adding the sequences obtained from GenBank to the alignment from [23] using the “add” function in MAFFT. Since the [23] reference alignment included more than one sequence for many species, we selected the sequence with the fewest gaps and ambiguous bases (N) for each species. The resulting alignment was visually inspected and sequences that did not align were reverse complimented and aligned again. We removed three species since their sequences were too short and contained mostly highly variable regions, making their alignment unreliable.

The alignment was then used to create a phylogenetic tree in RaxML version 7.4.2 [25] using 500 rapid bootstrap replications under the GTRCAT model.

#### Fungal body size and spore output

We combine our spore size database together with data from Hart and Reader [26–28] to test the trade-off of spore output and spore size, as well as the allometric relationship of these variables to fungal body size. We chose this experiment because (1) it is one of the few instances where the sporulation dynamics and total colony size of a phylogenetically diverse set of AM fungal species are reported; and (2) each variable was measured with the same protocols across all species. The data correspond to a greenhouse experiment where 14 AM fungal species were grown in pots with a single plant host. Four plant species were used as hosts for each of these 14 fungal species in the experiment (*Poa annua, Poa pratensis, Plantago major,* and *Plantago lanceolata*), with five replicates for each host-AM species combination. Each single host was inoculated with a standardized amount of AM inoculum and kept under controlled conditions (e.g., artificial light and low-P fertilization) for a total of 12 weeks. We used the data of root AM fungal colonization reported as micrograms of ergosterol per gram of dry root and soil colonization reported as meters of hyphae per gram of dry soil. Subsequently, we transformed ergosterol values to common units of length (meter) using the conversion parameters provided in Hart and Reader [26]:

$${{AMF}}\,{{root}}\,{{ergosterol}}\,\left( {\mu g} \right) = 0.4\,{{Hyphal}}\,{{length}}\,\left( m \right) + 0.18$$

We used ergosterol values (as opposed to the more traditional root colonization percentage based on visual counting) because it allowed us to estimate total fungal AM fungal body size as the sum of the colonization from both roots and soil in common length units. That is, we define fungal body size as the total mycelium [29]. We acknowledge that ergosterol might be a problematic biomass proxy for some AM fungi [30], but, at least in the case experiment of Hart and Read [26, 27], ergosterol and AM fungal colonization are well correlated [31].

Then, measures of hyphal length both from roots and soil were converted into biovolumes. We assume that hyphae are perfect cylinders of a constant radius (here we used a radius of 4 µm as a mid-size value within a range of 1–10 µm reported for AM hyphae [Smith and Read 2008]) and then using the conversion factor of Bakken and Olsen [32] of 1 cm^3^ hyphae = 0.23 g of hyphae (dry weight). We use this conversion factor because it has been empirically validated for the AM fungus *Funneliformis caledonium* by Olsson et al. [33] using two independent methods (see supplementary material for conversion factors). Spore output data correspond to spore densities (number of spores per gram of soil) from the same experiment, as reported in Hart and Reader [28]. Finally, for each of the 14 species, we used these spore output values with the corresponding spore size value in our database to calculate total mass allocated to reproduction (parameter *A* in Eq. (1)). That is:

Total mass allocated to reproduction (*A*) = spore output (*R*) * spore size (*W*_0_*)*

#### Offspring size of other taxa

We collected data from functionally analogous propagule units (offspring) from other microscopic soil fungi, seed plants (angiosperms), and birds for visual comparisons of the distribution of offspring size of AM fungi. For microscopic fungi, we digitized asexual spore (conidia) size of soil ascomycetes reported in the Compendium of Soil Fungi (360 species) [34]. Using the same approach described above for AM fungi, we calculated spore size from the inner-range values and used it to calculate biovolumes. We used this compendium because it is a standard reference on soil fungal diversity in mycology, providing well-curated data for the most common filamentous fungi found in soil. For plants, we used data from the Seed Information Database of Kew Botanical Gardens on seed-bearing angiosperms (http://data.kew.org/sid/?_ga=2.73581714.1287366807.1501084977-1309187973.1501084964). This dataset provides measurements of seed size as mass for 34,390 angiosperm species and has served as a database reference to map the distribution of seed size in plant ecology (see ref. [2] for more details). For birds, we used data from the recent egg-morphology compilation of Stoddard et al. [1], which reports egg volume data for species belonging to all extant orders of birds and is based on digital images of eggs present in the Museum of Vertebrate Zoology (University of California, Berkeley) database (1400 species) (see reference for details on data extraction and volume calculations).

### Statistical analysis

#### Phylogenetic conservatism

We tested whether AM fungal spore volume carried a phylogenetic signal, i.e., whether it differed from random trait variation expected under Brownian motion, using Pagel’s lamda (*λ*) [35]. This test has been shown to perform best compared to other tests for phylogenetic signal [36]. Given the dimorphic nature of some species in the Ambisporaceae, we performed two separate tests using either “acaulosporoid” or “glomoid” spore types for those species. We used the 'phylosig’ function in the package ‘phytools’ [37] in R [38].

#### Life history trade-offs

All statistical analyses were conducted with the logarithms of the following variables: spore size (as spore biomass), spore output (as number of spores), total resources allocated to spore production (as the product of spore output and spore size), fungal total biomass (as the sum of colonization in roots and soil), and total length of the extraradical mycelia. These last two variables allowed us to control for total fungal size when analyzing the allometric relationship between spore output and spore size. We used logarithms for two reasons: First, since we expect all relationships tested to follow a power function (Eqs. 1 and 2), using their logarithms linearizes the relationship, allowing the use of linear correlation to detect a trade-off. We also corrected for phylogenetic relatedness using the ‘PIC’ function from the ‘picante’ package [39] when non-corrected variables were significant. Doing so allowed us to determine whether the trade-off is the result of strong physical constraints acting on each species independently—regardless of their phylogenetic relatedness—or if it is a trait covariation pattern that resulted from limited evolutionary events that have been retained through lineages in the phylogeny.

Second, for the specific case of the trade-off between spore size and spore output, using logarithms allows us to estimate parameter *b* in Eq. 1 as a slope. We are interested in this parameter because it allows us to identify differences in patterns of resource allocation to spore production across species. If *b* = −1, spore size and spore output are inversely proportional to each other: increases in spore size translate into proportional reductions in spore output. This indicates that total resource allocation-to-spore production remains constant; in other words, resources are just partitioned differently into a few large spores or many small spores. If, on the other hand, −1 < *b* < 0, this would show that while variables are negatively correlated, they deviate from inverse proportionality: increases in spore size translate into less than proportional reductions in spore output. If this is the case, total resource-allocation-to-spore production would also result in an increase in spore size (larger-spored species would need to allocate more resources to produce a considerable number of large-sized spores). To estimate this slope, we used both linear regression and standardized major axis (SMA) regression using phylogenetically uncorrected variables. We did this because if some species allocate more resources to spore production than others, this allocation represents a real difference—regardless of how strongly reproductive traits co-vary with phylogeny. SMA slope calculations and their testing for significant deviation from −1 were done using the ‘smatr’ package [40] in R.

## Results

### Spore size distribution

**Fig. 1:**
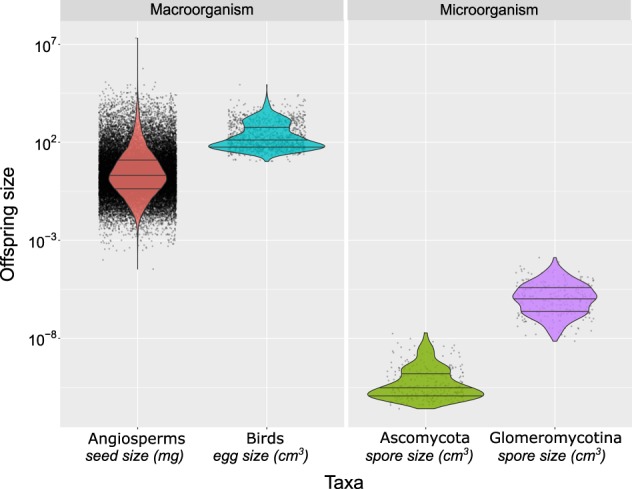
Comparison of offspring size variation across different taxa. Violin plots for comparison of the variation in propagule (offspring) size among AM fungi (294 species) with other common soil fungi in the Ascomycota (360 species); angiosperms (34390 species) and birds (1400 species). Each dot represents a species; violin plot width represents the data density at each level of offspring size, and lines within the plots depict the median and the first and third quartiles

Spore-size distribution was consistent and did not change when diameters were calculated using the inner or outer ranges (Fig. S1). Thus, in our subsequent analysis, we used spore-size diameter estimates calculated from inner-range means because we believe that these inner-range values are more representative of the spore size of a given species. This spore size distribution shows two main points (Fig. 1). First, that AM fungal spores are, indeed, big. In fact, when compared to soil fungi in the Ascomycota, there is almost no overlap between the two, and, on average, AM fungal spores are 1000 times larger (Fig. 1). Second, the range in size is large within AM fungi, spanning around four orders of magnitude. As a relatively species-poor group, this level of variation is comparable to that of bird’s eggs and half of that observed in angiosperm seeds (Fig. 1).

### Spore size distribution through the phylogeny

**Fig. 2:**
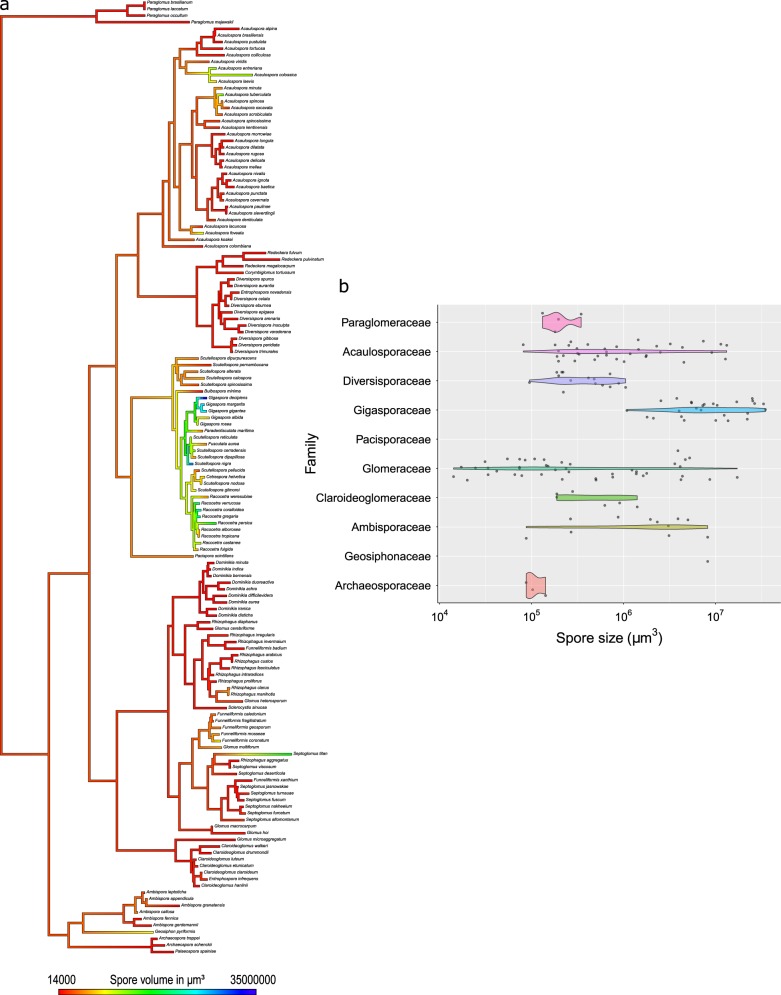
Distribution of spore size within the Glomeromycotina. **a** Variation in spore size through the AM fungal phylogeny. Differently colored branches of the phylogenetic tree indicate the spore volume values from the smallest values in red to the biggest in blue (this tree includes only the “glomoid” spore type for the Ambisporaceae, a near identical tree using “acaulosporoid” type is provided in the supplementary material); **b** violin plots showing the distribution of spore size variation within each family for species included in the phylogenetic tree. Each jittered dot represents an individual species (Paraglomeraceae = 4 species, Acaulosporaceae = 34 species, Diversisporaceae = 15 species, Gigasporaceae = 27 species, Pacisporaceae = 1 species, Glomeraceae = 43 species, Claroideoglomeraceae = 6 species, Ambisporaceae = 6 species, Geosiphonaceae = 1 species, and Archaeosporaceae = 3 species); violin plot width represents the data density at each level of offspring size

Early diverging AM fungal groups (families Paraglomeraceae and Archaeosporaceae) show consistently mid-to-low spore sizes (around 10^5^ µm^3^), while extreme values and larger ranges appear in more recent/diverse AM fungal lineages. For example, the family Gigasporaceae is made up of species that consistently exhibit large spore sizes (10^6^–10^7^ µm^3^), including the species with the largest spore size in the dataset (*Gigaspora decipiens*). The Glomeraceae is the most diverse taxon and has the largest range in spore size (three orders of magnitude, 10^4^–10^7^ µm^3^) and includes the species with smallest spore size in the dataset (*Glomus microaggregatum*). The Acaulosporaceae and Ambisporaceae are also diverse and exhibit a range of two orders of magnitude (10^5^–10^7^ µm^3^) (Fig. 2). In the case of the Ambisporaceae, this variability is partly due to the fact that some of the species produce two spore types with distinct sizes: the smaller “glomoid” and the bigger “acaulosporoid” type. This pattern in spore size distribution is reflected in the estimate of phylogenetic signal, which detected a strong significant difference from random trait variation expected (*λ* = 0.67, *P* < 0.001). This result holds up regardless of which spore type is included for the six dimorphic species in the Ambisporacae (Fig. S2).

### Reproductive allocation patterns

**Fig. 3:**
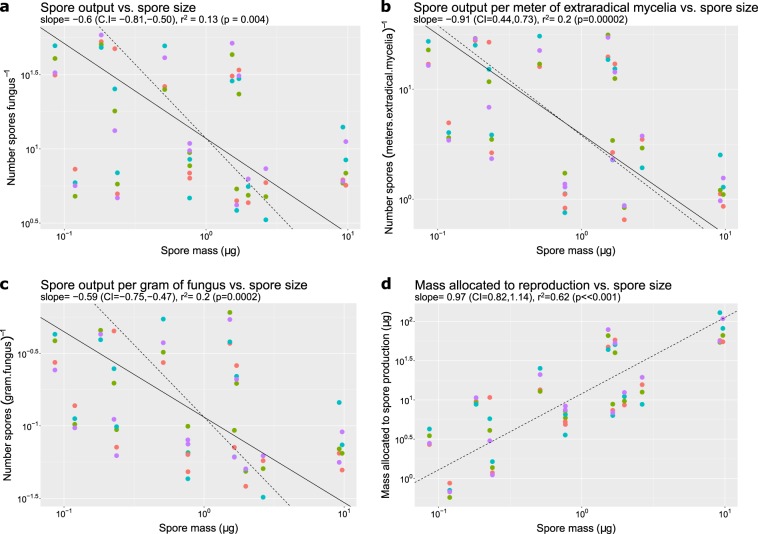
Spore size and spore output relationship. The solid line represents the SMA regression slopes of the analysis of 14 AM fungal species for which we could obtain reproductive allocation data [26–28]. The dotted line is the theoretical expectation of inverse proportionality for comparison. Each point represents an AM fungal species growing with a host. Different color points indicate different host species (red: *Plantago lanceolata*, green: *Plantago major*, blue: *Poa annua*, and purple: *Poa pratensis*). SMA slopes are reported together with their confidence intervals (CI) on the subheadings. Figures a to b differ on the way we correct the spore output–spore size relationship for fungal body size: **a** no correction for body size; **b** correction based only on the length of the extraradical mycelia; **c** correction based on total mass of fungal body size (both intra- and extraradical mycelia)

**Fig. 4:**
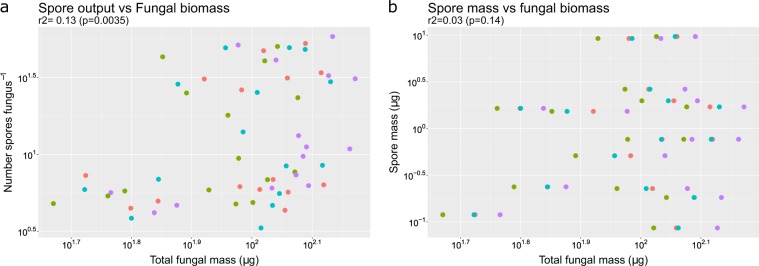
Scaling relationship of offspring size and offspring output to total fungal body size. Each point is an AMF species from which we could obtain reproductive traits and total fungal body size [26–28]. Different color points indicate different host species (red: *Plantago lanceolata*, green: *Plantago major*, blue: *Poa annua*, and purple: *Poa pratensis*)

The analysis of the 14 AM fungal species for which we could obtain reproductive allocation data [26–28] revealed four allocation-to-reproduction patterns via spores (Fig. 3; since both ordinary linear regression and SMA led to qualitatively similar results, only SMA slopes are presented as linear regression and always underestimate the slope values [41]). First, there is a significant negative correlation between spore output and spore size, which suggest that there is a trade-off between spore output and spore size (large-spored species produce fewer spores than small-spored species) (Fig. 3a–c). Second, the slope of this relationship is significantly higher than −1 when we control for total fungal biomass but is not significant when we control for total extraradical mycelia, indicating that this trade-off strongly reduced the fitness of the extraradical mycelia in terms of length, however, it is not strong enough to follow inverse proportionality in terms of biomass allocation. In other words, the fitness of the individual hyphae in the soil is strongly reduced (i.e., fewer spores are produced per meter of hyphae in soil [Fig. 3b]), but per gram of fungal mycelia, larger-spored species still allocate a considerable amount of biomass to produce a relatively high number of spores in the soil compared to their small-spored counterparts (Fig. 3c). Third, there is a positive correlation between the total amount of resources allocated to spore production and spore size. This suggests that in order to sustain the substantial spore output of large spores, larger-spored AM fungal species invest more resources into reproduction via spores than small-spored species (Fig. 3c). Finally, when analyzing the variation of these two reproductive traits (spore output and spore size) with the variation in total fungal body size (total biomass of mycelia), only spore output correlates with fungal size. This indicates that, while the larger mycelia are associated with greater total spore production, there is no evidence that supports the claim that large-spored species produce consistently large mycelia (Fig. 4).

## Discussion

### Ecological meaning of offspring size in AM fungi

Understanding the diversity of successful reproductive strategies in fungi is a major challenge. At first glance, the patterns we show here suggest a paradox for these fungi. On the one hand, the trade-off between spore output and spore size we show here (Fig. 3) suggests that AM fungal spores are costly and thus increases in individual spore size translate into higher energy and resource demands, which results in reduction of fitness for the parental mycelia. This explanation is not only consistent with the size–offspring output relationship seen in other taxa-like plants [42] but is congruent with the differential sporulation dynamics observed for five Glomeraceae and two Gigasporaceae species growing in vitro [43] and cytochemical analysis of spores. That is, AM fungal spores are made up of metabolically costly complex compounds such as lipids (comprising up to 95% of the spore C-pool), and storage carbohydrates [44] packed in semi-membranous vacuoles [45]. In addition, the multilayered wall of the spore is formed out of costly recalcitrant polymers including chitin (up to 47% of the wall) [46], melanin [47], glucans [48, 49], and sporopollenin [50].

On the other hand, AM spore size is a highly variable trait both overall and within specific clades, showing a comparable magnitude of variation seen in some macro-organisms (Fig. 1), suggesting that species with a range of spore sizes are competitive in natural ecosystems. As with the evolution of many traits in macro-organisms [51], there are clades that have distinctive values for spore size throughout the phylogeny. This variation would suggest that either different species are under distinct selection pressures and, thus, reflect distinct ecology [52] that, as we show, does not seem to depend on fungal body size (Fig. 4b), or trade-offs elsewhere in fungal life history equalize the fitness differences. As we discuss below, several selection pressures can be hypothesized for the maintenance of this variation, such as selective predation and dispersal or spore size-dependent germination and early colonization on heterogeneous environments.

Solving this paradox—maintenance of large offspring size variation despite fitness costs—requires more information that is not currently available for AM fungi. Specifically, reproductive ecology models for mammals and plants [4] take into account correlations of offspring size with length of reproductive lifespan, dispersal mode, and survival rates of offspring through lifespan (from dispersion to establishment as an adult).

For instance, a positive correlation between spore size and reproductive lifespan would “even out” fitness differences among large- and small-spored species. In fact, such correlation could explain why this trade-off is not strong enough to create inverse proportionality in terms of biomass allocation (−1 < *b* < 0, Fig. 3c). Because AM fungi are iteroparous with indeterminate growth, the relative allocation to spores versus hyphal growth is likely related to the life history of the species. This is the case for other indeterminate growth, iteroparous organisms such as perennial plants (indeed, the trade-off between seed size and seed output disappears when the entire plant reproductive lifespan is taken into account [42]). This positive correlation of offspring size and length of reproductive output is also congruent with the colonization patterns of some AM fungal species: large-spored species rely either exclusively or at least preferentially on spores as propagule units to colonize new hosts, in contrast to small-spored species, which can use hyphal fragments or colonized roots [53, 54]. Given these constraints, it makes sense for larger-spored species to have longer reproductive lifespans to compensate for their high cost. Indeed, when comparing the lifecycle under in vitro conditions of three species in the Glomeraceae and three species in the Gigasporaceae, species in the latter kept producing spores longer, even after the host was dead [43]. If this pattern holds true across a wider range of taxa, both large- and small-spored species could have similar spore outputs in their entire lifetimes, which would cancel out the trade-offs we show here that looked at spore production in a given window of time.

Likewise, dispersal mode is a factor that in some contexts explains offspring size variation. In plants, wind-dispersed species have, on average, smaller seeds than animal-dispersed ones [55]. Similarly, in macroscopic fungi, wind dispersion also seems to play a major role in the ecology and evolution of spore size [15, 56]. For AM fungi, wind dispersal, although recorded, seems unlikely as a major factor determining spore size, given their belowground production [57]. In contrast, other dispersal mechanisms, such as animals, might play a major role in AM fungi [58].

Finally, spore size, via differential allocation of resources, may influence offspring survival rates as it has been shown in the macro-world [6]. Unfortunately, to our knowledge, there is no across-AM fungal-species survival rate data throughout their lifetime (from dispersal to establishment to competition). The observation that larger-spored species can search longer (in space and time) for a host than small-spored species (reaching up to a 50-cm mycelium length after 20 days of growth in the case of the large-spored *Gigaspora margarita* [59]) supports the idea of a positive correlation between spore size and offspring survival. Alternatively, spore size survival relationship can depend on distinct abiotic and biotic conditions (e.g., drought or palatability to fungivores [60]), as has been shown in plant seeds [10]. Along these lines, an important factor to consider for AM fungi is host identity: early AM host colonization may require different amounts of spore resources depending on host identity (e.g., for the formation of hyphopodia). If this context-dependent survival is also coupled with distinct spore germination cues, there could be enough environmental heterogeneity to maintain large spore variation. Similarly, spore size could also influence establishment during competition among germlings. Little evidence is available on this topic. Two studies in which AM fungal germlings were placed in competition for the same host, one in an in vitro system [61] and the other a greenhouse pot study [62], showed that germlings from the species with the largest spore clearly outcompeted the others. In neither of the studies was spore size even considered as a factor explaining the competitive outcomes, despite the fact that in the in vitro study (two-species competition), the winning species had spores three times larger than the loser; while in the pot experiment (three species), the winning species had spores three to nine times larger than the other two competitors.

### Comparison with the reproductive ecology of other microorganisms

To what extent do the trends observed in AM fungi extend to other microorganisms? This question is hard to answer given the limited data and empirical tests on the causes and consequences of offspring size variation in microorganisms. For example, to our knowledge, there are no explicit tests of the relationship between offspring size and offspring output either for single-celled or filamentous microorganisms. While this relationship may not be relevant to single-celled species that reproduce via binary fission (which eliminates variation in offspring output variation), it could be used to understand the reproductive ecology of single-celled microorganisms that reproduce via multiple fission and of filamentous microscopic fungi and bacteria (e.g., actinobacteria) that reproduce via spore production. We think that studies aiming to address this allometric relationship can exploit the existing protocols and technology for microbial size measurements. Such methods span simple size class differentiation through sieving methods or via chemical gradients (a method that has already been successfully used to measure soil bacterial and archeal cell size from the field [63]), or using more sophisticated flow cytometry [64].

The relationship between offspring size and adult size has received relatively more attention. In the context of cell size regulation in bacteria and in archaea, it is well documented that daughter-cell size scales isometrically to the size of adult cells [65]. Similarly, Caval-Holme et al. [13] found that embryo size of species in the foraminifera (Protista) scales positively with adult cell size (although the relationship is less tight than in bacteria). The patterns of these single-celled microorganisms contrast with the lack of correlation between spore size and fungal body size we report here with AM fungi (Fig. 4b), as well as the weak correlation of spore size and sporocarp size reported for macroscopic fungi [66]. One possible explanation is that single-celled microorganisms, as unitary organisms with determinate growth, have strong scaling constraints that limit offspring size variation, while filamentous microorganisms, given their indeterminate, modular growth, do not. This comparison is analogous to the ones observed in the macro-world: the offspring size of mammals (unitary organisms) is tightly correlated with adult size, while for offspring size of plants (modular organisms) the relationship is weaker [4].

## Concluding remarks

Our results revealed a potential paradox in the reproductive ecology of AM fungi: large spore size variation is being maintained in the phylogeny but at the same time there is a trade-off with spore output. This paradox justifies the need for comparative studies across a variety of AM fungal taxa of survival rates of spores and germlings, competitive asymmetries among germlings (especially among those from different spore size classes), and sporulation lifespan dynamics of AM fungi as well as other morphological traits values (e.g., ranges of hyphal diameters, spore mass estimations). Additionally, intraspecific spore variability (which can be high, as reported for some species [Bever and Morton, 1999]) needs to be better documented to help understand how and why this trait has evolved (as well as to calibrate the use of spore size data in ecological studies); for example, testing whether different plant species or soil types influence the evolution of spore size for a given species. Although technically challenging, we think that obtaining such data is achievable in the near future, given the existence of culture collections and movements toward standardized approaches to generate trait data for fungi [68–70].

Beyond the specific case of AM fungi, we hope our work sparks new interest in addressing reproductive ecology questions in the microbial world. Here, we used pre-existing concepts from the macro-world to better understand the ecology and evolution of reproductive traits of microbes. However, this approach is not unlimited—some key features of microbial biology have no parallel in the macro-world. For example, one common feature of microbes (including the microbes studied here) is the evolution of symbioses, either with macro-organisms or with other microbes. None of the current macro-centric frameworks include host influences on the evolution and ecology of reproductive traits of their microbial symbionts. Already Garrett, by 1973 [71], proposed that the spore content of plant pathogenic fungi is the result of evolution to differences in host infection strategies: resources present in large spores reflected infection of healthy hosts, while species with small spores would be restricted to infect senescent or highly stressed hosts. Addressing this and other unique microbial biology features will require the development of new conceptual frameworks.

## Electronic supplementary material

Dataset Conidia size of soil ascomycetes

Dataset Spore size AMF

Calculations, data conversion factors and supplementary figures
