# Supplementary material for: Bridging reproductive and microbial ecology: a case study in arbuscular mycorrhizal fungi
Source: ISME J. 2018 Nov 30;13(4):873–84. doi: 10.1038/s41396-018-0314-7 (PMC6461870; doi:10.1038/s41396-018-0314-7)
Supplement: Supplementary file 3 — Calculations, data conversion factors and supplementary figures [file 41396_2018_314_MOESM3_ESM.docx]

**Bridging reproductive and microbial ecology: a case study in arbuscular mycorrhizal fungi**

Carlos A. Aguilar-Trigueros^1,2*^, Stefan Hempel^1,2^, Jeff R. Powell^3^, William K. Cornwell^4^, Matthias C. Rillig^1,2^

*^1^ Freie Universität Berlin, Institute of Biology, D-14195 Berlin, Germany*

*^2^ Berlin-Brandenburg Institute of Advanced Biodiversity Research (BBIB), D-14195 Berlin, Germany*

*^3^ University of Western Sydney, Hawkesbury Institute for the Environment, Penrith NSW 2751, Australia*

*^4^ Evolution and Ecology Research Centre, School of Biological, Earth and Environmental Sciences, Univ. of New South Wales, Sydney, NSW, Australia*

*****Corresponding author: Carlos A. Aguilar-Trigueros, Altensteinsstrasse 6, Freie Universität Berlin, Institute of Biology, D-14195 Berlin, Germany; phone number: 49 (0)30 838-53143; e-mail: calgit@gmail.com

**Supplementary material**

Spore volume calculations

For spores reported as primarily “globose”, where only one diameter range is provided in the description, spore volume is calculated as a sphere. For this, we assume that all three diameters that define a sphere have the same value. For example, in the entry of the AM fungus *Acaulospora alpina:*

Given that only one diameter is provided, we calculate a single mean diameter for the species (values are in micrometers):

| Species name | dim1.min2 | dim1.min | dim1.max | dim1.max2 | dim2.min2 | dim2.min | dim2.max | dim2.max2 |
| --- | --- | --- | --- | --- | --- | --- | --- | --- |
| Acaulospora_alpina | 53 | 65 | 85 | 97 | 53 | 65 | 85 | 97 |

Diameter = (65+85)/2 = 75

Then we use the formula:

$$Spore volume={(Diameter}^{3}*\pi)/6$$

Which gives for *A. alpina*:

Spore volume = ((75)^3^ * π) = 2.2 x 10^5^ µm^3^

For spores reported as primarily “subglobose”, where two different diameter ranges are provided in the description, spore volume is calculated as a prolate spheroid. This means two things: first, that the smallest diameter will be considered the "equatorial axis" and the largest is the "polar axis"; second, that there are two equatorial axes of the same length (this means that we duplicated the smallest diameter). In this way, AMF subglobose spores are viewed as spheroids similar to an “american football”. For example, in the case of the AM fungus *Claroideoglomus lamellosum:*

Given that two different diameter ranges are provided; we calculate a mean value for each (values are micrometers)*:*

| Species name | dim1.min2 | dim1.min | dim1.max | dim1.max2 | dim2.min2 | dim2.min | dim2.max | dim2.max2 |
| --- | --- | --- | --- | --- | --- | --- | --- | --- |
| Claroideoglomus  lamellosum | 98 | 106 | 142 | 142 | 122 | 122 | 162 | 162 |

- Diameter 1: (106+142)/2 = 124
- Diameter 2: (122+162)/2 = 142

Thus, in this case diameter 1 is the equatorial axis and diameter 2 is the polar axis.

Then, we use the formula:

$$Spore volume={((Equatorial axis)}^{2}*Polar axis*\pi)/6$$

Which gives for *C. lamellosum*:

Spore volume = ((124)^2^*142*π)/6 = 1.14 x 10^6^ µm^3^

Conversion from spore volume to spore mass

Beilby and Kidby (1980) report in their table 1 that a single spore of *Funneliformis caledonium* (syn. *Glomus caledonius*) weighs in between 0.99 to 2.06 µg depending on soil conditions (these values were obtained after measuring 1000 spores of the fungus). We took the average of these two values as a reference for spore mass for this fungus. This means:

Spore mass of *F. caledonium* = 1.5 µg

Then we use the spore volume of *F. caledonium* in our database to calculate the weight of a cubic micrometer of AM fungus:

Spore volume of *F. caledonium* = 4.11 x 10^6^ µm^3^ = 1.5 µg

Then,

1 µm^3^ of *F. caledonium* = 3.64 x 10^-6^ µg

This value was used as a conversion factor to convert spore volume to spore mass in testing the trade-off between with spore output and the allometric relationship to fungal body size.

Conversion from hyphal length to fungal body size (as biomass)

We define fungal body size as the sum of root and soil colonization of a AM fungus given in biomass units. However, in the papers of Hart and Reader (2002a,b) that we used to test the allometric relationships of spore size and offspring output to fungal body size, fungal colonization is given as hyphal length in meters. To convert these hyphal length values to biomass units, we first transformed hyphal length values to volume, for which we assume that hyphae are perfect cylinders (Bååth and Söderström 1979) with a constant radius of 4 µm (or 4 x 10^-6^ m). Thus, using hyphal length values (in meters) we calculated hyphal volume as:

$$Hyphal volume {(m}^{3})=Hyphal length (m)*\pi*{(4x{10}^{-6}(m))}^{2}$$

Second, we used the parameters for conversion from hyphal volume to fungal biomass reported in Bakken and Olssen (1983):

- 1 cm^3^ hyphae = 1.09 g hyphae (fresh weight)
- 1 g hyphae (fresh weight) = 0.21 g hyphae (dry weight)

For example, a total hyphal length 5 meters will return 57.52 μg fungal body size as follows:

$$Fungal biomass \left( \mu g \right)= 5\left( m \right) \pi\left( 4x{10}^{-6}\left( m \right) \right)^{2} x \frac{{(100cm)}^{3}}{{1m}^{3}}x\frac{1.09 g Hyphae-Fresh weight}{{(cm}^{3}) hyphae} x\frac{0.21 g Hyphae-Dry weight}{1 g Hyphae-Fresh weight} x \frac{{10}^{6} \mu g}{1g}=57.52 micrograms$$

Fig. S1


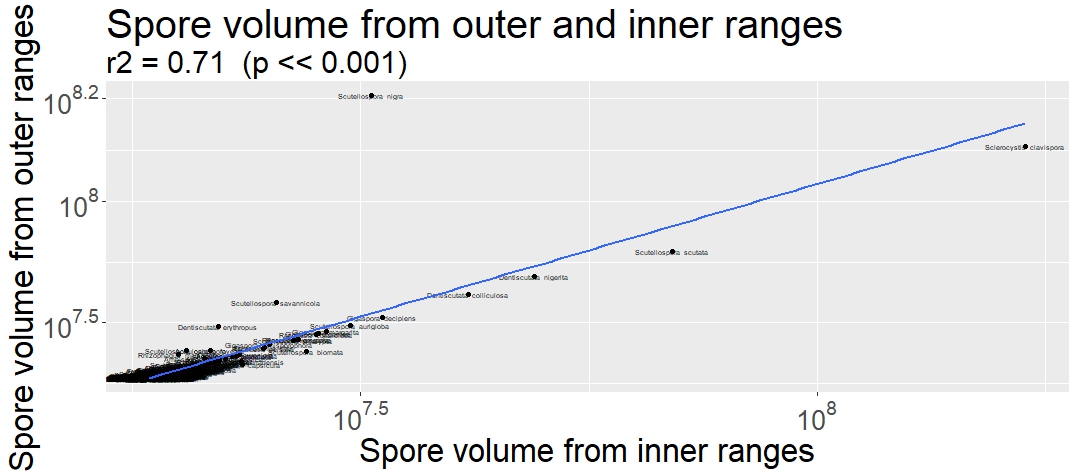


Fig. S1. Correlation between spore volumes calculated using mean diameter from inner ranges or outer ranges. Aside from the fungus Scutellospora nigra, the volumes are strongly correlated. In the case of *S. nigra,* we kept using the diameter from the inner range because it also corresponds to the mean diameter reported in the original description of the species by Nicolson and Schenck 1979.

Fig. S2. a) Variation in spore size through the AM fungal phylogeny (this tree includes only the “acaulosporoid” spore type for the Ambisporaceae).


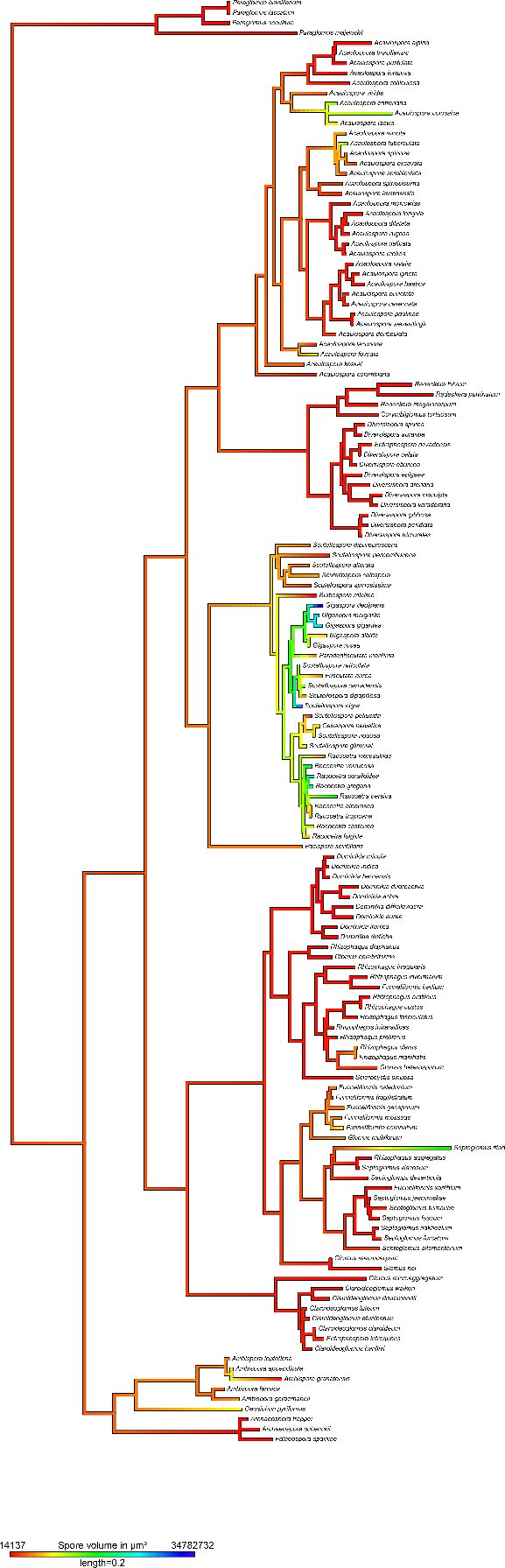


**References**

1. Beilby, J. P. & Kidby, D. K. Biochemistry of ungerminated and germinated spores of the vesicular-arbuscular mycorrhizal fungus, Glomus caledonius: changes in neutral and polar lipids. *J. Lipid Res.* **21**, 739-750 (1980).
2. Hart, M. M. & Reader, R. J. Does percent root length colonization and soil hyphal length reflect the extent of colonization for all AMF? *Mycorrhiza* **12**, 297-301, doi:10.1007/s00572-002-0186-5 (2002).
3. Hart, M. M. & Reader, R. J. Taxonomic basis for variation in the colonization strategy of arbuscular mycorrhizal fungi. *New Phytol.* **153**, 335-344, doi:10.1046/j.0028-646X.2001.00312.x (2002).
4. Bååth, Erland & Söderström, Bengt*.* The Significance of Hyphal Diameter in Calculation of Fungal Biovolume. *Oikos* **33**, 11-14, doi:10.2307/3544505 (1979).
5. Bakken, L. R. & Olsen, R. A. Buoyant densities and dry-matter contents of microorganisms: conversion of a measured biovolume into biomass. *Appl. Environ. Microbiol.* **45**, 1188-1195 (1983).
6. Nicolson, T.H. & Schenck, N.C. Endogonaceous mycorrhizal endophytes in Florida. *Mycologia.* (1979).
